# Supplementary material for: Hypoxia-Induced Epithelial-to-Mesenchymal Transition in Proximal Tubular Epithelial Cells through miR-545-3p–TNFSF10
Source: Biomolecules. 2021 Jul 15;11(7):1032. doi: 10.3390/biom11071032 (PMC8301867; doi:10.3390/biom11071032)
Supplement: Supplementary file 1 [file biomolecules-11-01032-s001.zip › biomolecules-1250631-supplementary.pdf]

Table S1. Target sequence of the materials utilized in the study.

**hsa-miR-545-3p**	5′d TCAGCAAACATTTATTGTGTGC 3′
**hsa-miR-1269a**	5′d CTGGACTGAGCCGTGCTACTGG 3′
**hsa-miR-4474-3p**	5′d TTGTGGCTGGTCATGAGGCTAA 3′
**hsa-miR-5579-3p**	5′d TTAGCTTAAGGAGTACCAGATC 3′
**hsa-miR-1266-5p**	5′d CCTCAGGGCTGTAGAACAGGGCT 3′
**hsa-miR-3613-3p**	5′d ACAAAAAAAAAAGCCCAACCCTTC 3′
**hsa-miR-33b-5p**	5′d GTGCATTGCTGTTGCATTGC 3′
**hsa-miR-190a-3p**	5′d CTATATATCAAACATATTCCT 3′
**hsa-miR-1277-3p**	5′d TACGTAGATATATATGTATTTT 3′
**hsa-miR-33a-5p**	5′d GTGCATTGTAGTTGCATTGCA 3′
**hsa-miR-219a-5p**	5′d TGATTGTCCAAACGCAATTCT 3′
**miRIDIAN microRNA Human has-miR-545-3p-Mimic**	UCAGCAAACAUUUAUUGUGUGC
**Homo TNFSF10**	Forward GCTCCTGCAGTCTCTCTGTG
	Reverse ACGGAGTTGCCACTTGACTT
**Homo Glyceraldehyde-3-phosphate dehydrogenase (GAPDH)**	Forward GAGTCAACGGATTTGGTCGT
	Reverse TTGATTTTGGAGGGATCTCG

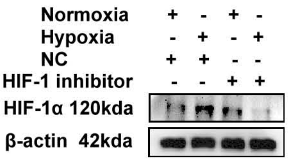
Normoxia + - + -
Hypoxia - + - +
NC + + - -
HIF-1 inhibitor - - + +
HIF-1α 120kda
β-actin 42kda
